# Patient satisfaction with virtual evaluation, diagnosis, and treatment of CRPS

**DOI:** 10.1080/24740527.2022.2063113

**Published:** 2022-06-03

**Authors:** Emma Loy, Anna Scheidler, Tara Packham, Heather Dow, Paul Winston

**Affiliations:** a Faculty of Medicine, University of British Columbia, Vancouver, British Columbia, Canada; b Faculty of Medicine, Dalhousie University, Halifax, Nova Scotia, Canada; c School of Rehabilitation Sciences, McMaster University, Hamilton, Ontario, Canada; d Canadian Association of Physical Medicine and Rehabilitation, Kingston, Ontario, Canada; e Division of Physical Medicine and Rehabilitation, University of British Columbia, Vancouver, British Columbia, Canada

**Keywords:** complex regional pain syndrome, CRPS, virtual care, physiatry, physical medicine and rehabilitation, telemedicine, telehealth, e-health

## Abstract

**Background:**

The COVID-19 pandemic has led to an increased reliance on virtual care in the rehabilitation setting for patients with conditions such as complex regional pain syndrome (CRPS).

**Aims:**

The aim of this study was to perform a quality improvement initiative to assess patient satisfaction and ensure that outcomes following virtual assessment, diagnosis, and treatment of CRPS with prednisone are safe and effective.

**Methods:**

An online survey was distributed to 18 patients with CRPS who had been seen virtually between March and December 2020 through a rehabilitation clinic and treated with oral prednisone. Thirteen participants completed the survey, which was designed de novo by our team to evaluate participant perceptions and satisfaction regarding the virtual care experience. Also included in the survey was a CRPS-specific validated patient-report questionnaire (Hamilton Inventory for CRPS: PR-HI-CRPS), which allowed participants to describe their specific symptoms and associated functional and psychosocial impacts, both previously (pretreatment baseline) and at the time of survey (posttreatment).

**Results:**

CRPS symptoms and related impacts were scored as significantly improved from baseline following treatment with prednisone. Likert scale results from survey responses related to patients’ experiences and satisfaction with the virtual care process were analyzed; the majority of patients reported satisfaction with a virtual appointment for evaluation of CRPS, as well as with subsequent treatment decisions based on virtual assessment.

**Conclusions:**

This quality improvement study suggests that virtual care is a potential option for a patient-accepted approach to overcoming challenges with in-person care imposed by the COVID-19 pandemic and could help inform future considerations in addressing geographic and patient-specific disparities in access to specialist care for CRPS.

## RÉSUMÉ

**Contexte**: La pandémie de COVID-19 a donné lieu à un recours accru aux soins de réadaptation virtuels pour les patients souffrant d’affections telles que le syndrome douloureux régional complexe (SDRC).**Objectifs**: L'objectif de cette étude était de réaliser une initiative d'amélioration de la qualité afin d’évaluer la satisfaction des patients et de veiller à ce que les suites à l'évaluation, au diagnostic et au traitement virtuel du SDRC par la prednisone soit sûres et efficaces.**Méthodes** : Un sondage en ligne a été distribué à 18 patients atteints de SDRC qui avaient été vus virtuellement entre mars et décembre 2020 par le biais d’une clinique de rééducation et qui avaient été traités par prednisone orale. Treize participants ont répondu au sondage, qui avait été conçu de novo par notre équipe, afin d’évaluer les perceptions et la satisfaction des participants à l'égard de leur expérience de soins virtuels. Le sondage comprenait aussi un questionnaire validé par le patient propre au SDRC, (le Hamilton Inventory for CRPS: PR-HI-CRPS), qui a permis aux participants de décrire leurs symptômes spécifiques, ainsi que les effets fonctionnels et psychosociaux qui y étaient associés précédemment (avant le traitement) et à la date de l'enquête (post-traitement).**Résultats**: Les notes accordées par les participants ont révélé que les symptômes du SDRC et leurs répercussions s’étaient significativement améliorés aprés le traitement par la prednisone. Les résultats sur l'échelle de Likert découlant des réponses aux questions de l'enquête sur l’expérience des patients et leur satisfaction à l'égard du processus de soins virtuels ont été analysés. La majorité des patients se sont déclarés satisfaits du rendez-vous virtuel pour l'évaluation du SDRC, ainsi que des décisions de traitement ultérieures basées sur l'évaluation virtuelle.**Conclusions**: Cette étude d'amélioration de la qualité suggére que les soins virtuels sont une option envisageable en tant qu’approche acceptée par le patient pour surmonter les difficultés liées aux soins en personne imposées par la pandémie de COVID-19. Elle pourrait donc aider à éclairer les considérations futures dans le traitement des disparités géographiques et les celles qui sont spécifiques aux patients dans l'accés aux soins spécialisés pour le SDRC.

## Introduction

Physician practice was abruptly altered when the COVID-19 pandemic was officially declared on March 11, 2020, by the World Health Organization. In Canada, states of emergency and shutdowns of nonessential businesses began on March 17, 2020. Within days, elective medical procedures and nonurgent treatments were postponed. Patients with acute medical events were still seen in hospital, but outpatient clinics were largely shuttered. As a result, a shift to reliance on virtual care for outpatient diagnosis and treatment occurred almost immediately.

Prior to the pandemic, virtual care in the rehabilitation setting had received increased consideration as an acceptable or even preferable alternative to an in-person appointment for some patients, such as those facing physical mobility challenges or residing in rural areas.^1^ Virtual care has been proposed to decrease travel time for patients, decrease overhead costs for physicians, and enhance geographical access to physiatrists.^2^ Our region already maintained a robust video telehealth program funded and remunerated equal to in-person visits. However, due to the unexpected rapid and widespread adoption of virtual care practices brought about by the pandemic in Canada, ongoing evaluation is necessary to efficiently maximize potential benefits, address known limitations, and identify barriers to positive patient outcomes.^3^

Though there has been some evaluation of the use of virtual care in rehabilitation medicine, we found that there are few studies that address virtual care for the evaluation and treatment of complex regional pain syndrome (CRPS). Our quality improvement study focuses on patients assessed and treated virtually for CRPS, a pain condition that affects a region of the body to a degree disproportionate to the inciting event. The syndrome is characterized by a constellation of signs and symptoms; pain and edema are often accompanied by sensory, vasomotor, sudomotor, motor, and/or trophic changes.^4,5^ The etiology and presentation of CRPS are heterogeneous. However, it is notable that fracture is the identified cause of CRPS in 42% to 44% of cases,^6,7^ with a higher incidence among women—up to two to four times that in men.^4,6^ The upper extremity is predominantly affected.^4^

After the pandemic lockdown commenced, a higher than typical percentage of new patient referrals was received by the physician investigator, which were directed to a rapid access triage program for suspected acute-onset CRPS. The referrals were largely initiated by surgeons who had treated patients with trauma in the hospital setting. Given lockdown restrictions, it became necessary to assess these patients virtually for signs and symptoms of CRPS and to determine whether they met the Budapest Criteria.^8^ Prior to the pandemic, this assessment would have been done in person. Patients were then treated with the clinic’s regimen of oral prednisone.^9^ Even after clinics reopened, many patients, due either to travel distance or to fear of COVID-19 exposure risk in health care settings, chose to continue with virtual assessment. It was therefore necessary to perform a quality improvement initiative to assess patient satisfaction and ensure that outcomes following virtual assessment, diagnosis, and treatment of CRPS with prednisone are safe and effective.

## Methods

### Study Participants

To meet inclusion criteria for this retrospective cohort study, participants had to fulfill the clinical Budapest Criteria^8^ and present with limited range of motion in more than one joint, as assessed by clinical examination. Additionally, they must have been prescribed a course of prednisone as per our clinic’s regimen and have undergone reassessment following completion of this treatment. To ensure that all cases were captured, a search was performed for the *International Classification of Diseases* code for CRPS (causalgia) in the electronic medical record of one physician for all cases between March 17, 2020, and March 17, 2021. All participants had been referred to this physician with a query of CRPS. They were triaged as urgent cases and booked for a virtual appointment. One of two platforms (FaceTime or Doxy.me) was used for each appointment, chosen based on patient preference and convenience. The physical examination was modified using telehealth methods from a previously published protocol (Table 1).^9^

**Table 1.** Detailed methods of assessing CRPS signs by physiatrist.

Sensory	To evaluate for hyperalgesia, the patient or their companion touched the affected limb with a sharp object and the patient noted whether an increased pain response was elicited. To evaluate for allodynia, the patient or their companion applied a light touch to the affected limb. Altered sensation/paresthesia was assessed by applying an identical force to both a CRPS-affected area and a corresponding non-CRPS-affected area on the contralateral extremity; the patient was then asked to comment on any perceived difference in sensation.
Vasomotor	Temperature asymmetry was assessed without specialized equipment. If a companion was present, that person was asked to touch both the patient’s CRPS-affected limb and contralateral non-CRPS-affected limb simultaneously to assess for temperature discrepancy. If unaccompanied by a companion, the patient was asked to stroke their face with both hands or arms simultaneously (all participants were affected by upper-limb CRPS). Skin color changes were determined by visual comparison between CRPS-affected and non-CRPS-affected areas.
Edema/sudomotor	Edema was evaluated by looking for signs of generalized swelling and lack of normal skin wrinkles; at the knuckles, for example. Sudomotor signs (sweating changes or asymmetry) were considered by observing and comparing sweat patterns at a CRPS-affected and a non-CRPS affected region. Assistance from the patient was requested if the video image was not clear.
Motor/trophic	Active and passive range of motion of all proximal and distal joints of the affected limb was evaluated, with assistance from a companion for some participants. Weakness, defined as decreased strength relative to the unaffected side, was evaluated using whatever materials were readily available to the patient. Reflexes were not tested.
Other	The physician evaluated for increased or decreased hair and nail growth by visually comparing CRPS-affected and non-CRPS-affected areas, confirming with the patient that differences observed were not due to a nonnatural cause (i.e., shaving only one limb or cutting nails on only one side of the body). Skin changes were defined as the presence of shiny skin, brawny discoloration, or other observed asymmetries.

### Survey Tool and Data Collection

This study was conducted as a quality improvement project to assess patient satisfaction with virtual diagnosis and treatment of CRPS and, according to Article 2.5 of the Tri-council Policy Statement, did not fall within the scope of research ethics board (REB) review. The local REB was consulted and recommended completing the Arecci Tool score, which produced a score indicating minimal risk. The REB also noted that the review of emergency transition to virtual care and its continuation were consistent with quality improvement investigation; thus, REB application and approval were deemed unnecessary. We were directed to follow the Qualitive Improvement (QI) registry instead. An anonymous online survey tool was developed by project team members and used for data collection. Survey questions were created de novo by our team and included fixed-choice and open-ended qualitative responses, assessing participants’ perceptions regarding various aspects of the virtual care experience. A second part of the survey included a 40-item CRPS-specific validated patient-report questionnaire (the Hamilton Inventory for CRPS: PR-HI-CRPS), describing the symptoms of CRPS, the impact of those symptoms on daily activities, and associated psychosocial impacts. Higher scores indicate more symptoms and greater impact on daily activities and psychosocial function.^10^ Though none of the questions on the PR-HI-CRPS were altered for this study, their order was adjusted to group items using the same scale anchors, because pretesting identified potential response errors in the online format when items were presented in their original (random) ordering. Participants completed the PR-HI-CRPS twice as part of the overall survey, first to recall their condition prior to accessing virtual care and then to describe their current status. Informed consent was obtained from all participants both verbally upon recruitment of participants and online prior to completion of the survey. Prior to providing consent, all participants were made aware that the results of this study would be published in an academic journal. All data collection occurred electronically via the Alchemer (www.alchemer.com, Louisville, CO, USA) web-based platform.

### Virtual Examination Methods

If the patient was accompanied by a companion, the companion was instructed on how to assess the patient for sensory disturbances or assist with range of motion. If alone, the patient was given instructions. See Table 1 for details.

### Treatment

This clinic’s typical prednisone regimen started with 60 mg followed by a taper of 5 mg per day until a dose of 20 mg was reached.^9^ Patients then remained on 20 mg for 1 week, followed by 15 mg for 1 week, and finally 10 mg for 1 week. A slightly modified decreased dose was chosen for elderly patients, adolescents, and patients with diabetes, typically starting at 40 mg before the taper, as described in previous studies.^9^ All patients were instructed to continue with their existing physiotherapy treatment.

### Data Analysis

Survey responses were exported to Excel for calculation of descriptive statistics, including frequencies and percentages for categorical data and means and ranges for continuous data. Responses from the PR-HI-CRPS were exported to STATA, Version 13 (StataCorp LLC, College Station, TX) for further analysis. Missing values for a single item at follow-up were noted for two participants; the baseline values were therefore imputed. The data for individual subscales (symptoms, function, and psychosocial impact summary scores) and total scores were converted to normalized values (/100) for ease of interpretation and were checked to ensure that they satisfied the assumption of normal distribution. Paired *t* tests were conducted comparing participants’ recall of pretreatment status with current status. Effect size (ES) for virtual care was calculated using Cohen’s *d* under the assumption of unequal variances, where ES < 0.02 was interpreted as a small effect and ES > 0.08 was considered to be a large effect.^11^

## Results

The survey was delivered to 18 patients who were diagnosed with CRPS virtually by the lead investigator. Of these, 13 (12 female and 1 male) completed the survey, yielding a completion rate of 72.2%. The average age of participants was 61 years (range 51–72). Geographically, 38% of participants lived in the same city as the physiatrist offering virtual appointments, 23% lived within a 2-h travel distance, and 38% lived further away.

**Figure 1.**
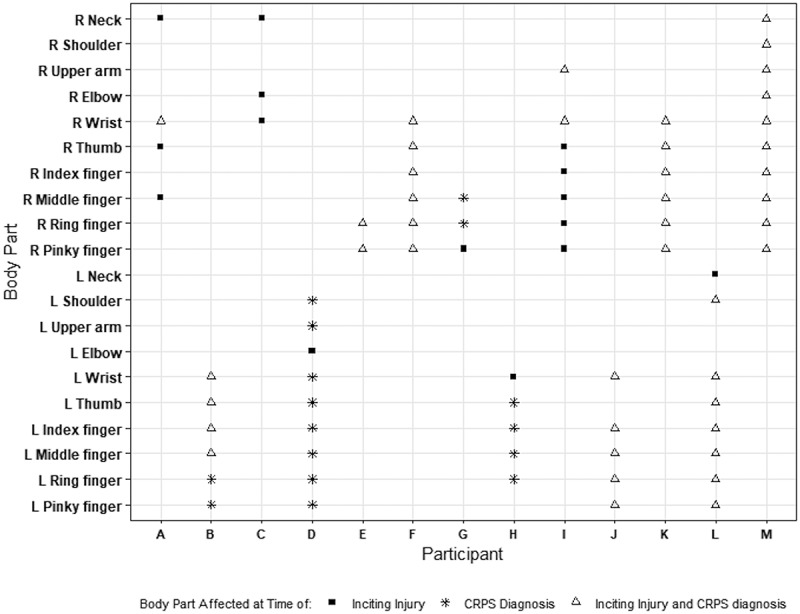
Body parts affected at original injury and at the time of referral for virtual evaluation of CRPS.

The study population included patients treated for CRPS of the upper limbs only. Nine reported an upper extremity fracture as the original injury, two were after carpal tunnel surgery, one was a fall on outstretched hand with a laceration, and one was a sprain. The specific locations of symptoms in the upper limb are reported in Figure 1. Following the original injury, 46% of participants developed symptoms affecting other parts of their upper limbs. For 69% of respondents, the affected limb felt hotter compared to the unaffected side, and for 15% the affected side felt colder than the unaffected side. Only one participant did not notice a change in temperature of the affected limb. Numbness was a complaint in 62% of participants.

Participants’ initial injuries occurred during a 12-month period between October 2019 and October 2020. Each participant had been referred for a virtual physiatry appointment, where a course of prednisone was initiated where appropriate. Appointments were conducted between March and December 2020. Sixty-two percent of participants were seen using the virtual platform Doxy.me and 38% using FaceTime. Patients’ prior exposure to virtual care is reported in Table 1. The average time elapsed from the date of the initial injury to the date of referral was 90 days (range 33 to 146 days). The average time elapsed from referral to virtual physiatry appointment was 15 days (range 3 to 28 days). One outlier (72 days) was excluded because the patient sought treatment elsewhere between their referral and appointment dates. Participants scored their symptoms and impact at baseline on the PR-HI-CRPS as an average of 60.5/100 (range 42.1–83.3, SD 13.3); total scores were normally distributed.

The proportion of patients who received hand therapy in any format (virtual, in-person, or a combination) before or after their assessment for CRPS is reported in Table 2.

**Table 2.** Quantitative patient responses to questionnaire.

	Virtual hand therapy, *n* %	In-person hand therapy, *n* %	Virtual and in-person hand therapy, *n* %	No hand therapy, *n* %
Patients receiving hand therapy *before* physiatry assessment for CRPS, *N* = 13	6 (46)	1 (8)	2 (15)	4 (31)
Patients receiving hand therapy *after* physiatry assessment for CRPS, *N* = 13	7 (54)	1 (8)	2 (15)	3 (23)
	None, *n* %	Mild, *n* %	Moderate, *n* %	Severe, *n* %
Return of symptoms after treatment, *N* = 11	4 (36)	4 (36)	3 (27)	0 (0)
	Had used telehealth previously, *n* %		Had not used telehealth previously, *n* %	
Patient exposure to telehealth prior to physiatry appointment, *N* = 13	4 (31)		9 (69)	
	Yes, *n* %		No, *n* %	
Would you recommend treatment of an injury similar to yours through virtual care?	10 (77)		3 (23)	
Would you have preferred an in-person appointment if possible?	7 (54)		6 (46)	
Did you encounter any barriers to engaging in virtual care? If yes, did you encounter the following: (a) Did not have a suitable device (b) Uncomfortable with the technology (c) Poor Internet connection (d) Other	2 (15) 0 (0) 0 (0) 1 (8) 1 (8)		11 (85)	

Eleven participants (85%) reported that it had been more than 4 months since the conclusion of their treatment with prednisone, and two (15%) were not yet 4 months posttreatment at the time of survey. Return of symptoms in patients four or more months posttreatment is reported in Table 1. Current CRPS symptoms and related impacts were scored as significantly improved from baseline, with a mean score of 27.5 (range 1.3–55.4, SD 17.0, mean change = 33, *P* < 0.001) and an effect size of *d* = 2.2. Full reporting of the PR-HI-CRPS scores is available in a supplemental table (Supplemental Table 1).

**Figure 2.**
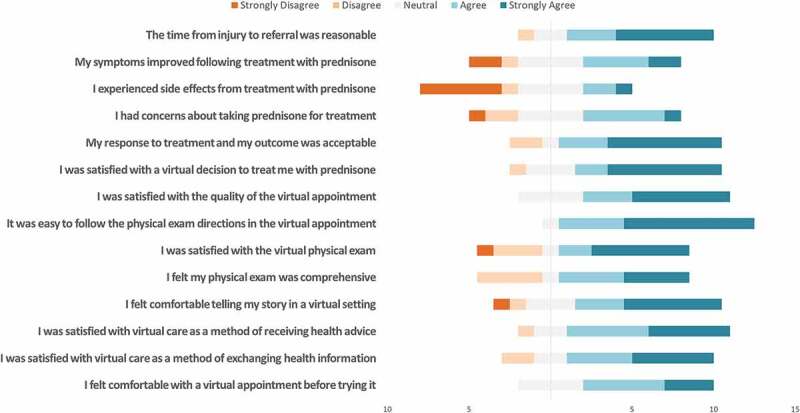
Participant responses to 5-point Likert scale questions about experiences with virtual care.

Likert scale results from survey questions related to patients’ experiences with virtual assessment for CRPS, as well as their experiences with the physician making the decision virtually to treat them with prednisone, are reported in Figure 2.

Participants provided qualitative responses to an open-ended survey question addressing their experience with virtual assessment and treatment prescribing. They were also invited to comment on their present condition following treatment with prednisone. Within their responses, ten participants reflected on the use of virtual care as an alternative to in-person appointments for CRPS management. Representative excerpts are shown in Table 3.

**Table 3.** Compilation of participants’ qualitative responses.

Excerpts from participant free-text responses regarding the virtual care experience
“I am grateful for the treatment but believe a virtual diagnosis was not enough information for [the doctor] to truly know my condition. It was also difficult because we are extremely rural and our connection kept stalling and cutting out.”
“Virtual follow-up has been very successful, easy, and convenient. I feel very fortunate to have this care.”
“Seeing the doctor virtually is much more like actually having a visit than talking on the phone. If I cannot see the doctor, virtual is satisfactory/preferred.”
“I found virtual calls very reassuring and a big help.”
“It was very important to have access to professional and personalized treatment after my injury, especially with the background pandemic disrupting normal access.”
“I was very impressed with the virtual service. The assessment and treatment were great. I was fully confident in [the doctor’s] ability to assess me virtually. I would like to keep doing it this way.”
“It worked very well. It makes sense in that people do not have to travel from all over the place to see specialists.”
“My preference would have been an in-person consult/initial exam, but I understand that due to COVID it wasn’t possible.”
“I think telehealth is an amazing resource for remote communities.”
“So glad that I was referred to [the doctor] and virtual treatment was available. I highly doubt that I would have been able to get treatment had virtual consultation not been an option.”

## Discussion

The COVID-19 pandemic necessitated a transition from in-person to virtual assessments for patients with conditions like CRPS. Assessing patient satisfaction and experiences with virtual care is essential in determining whether it is an appropriate modality for diagnosis and treatment of specific conditions.^12^ Our quality improvement study explores participant characteristics and barriers to virtual care, overall patient satisfaction, accessibility and comfort with virtual care, as well as satisfaction with specific elements of virtual care such as the timeline from referral to appointment, the virtual physical exam, and the virtual decision to treat suspected CRPS with prednisone and hand therapy.

Recent studies have demonstrated that age is not a barrier to engaging in virtual care.^13^ Our study is concordant with these findings. The average age of participants in this study was 61 years (range 51–72). No respondents reported feeling uncomfortable with the technology, and all participants had access to an appropriate device. Barriers to engaging in virtual care were minimal, with only one participant reporting difficulty due to a poor Internet connection and one feeling that they could not adequately express their story in a virtual setting. It is possible that the five people who did not complete our survey encountered barriers to using virtual tools and that those unable to engage in virtual care were excluded from accessing an initial virtual care appointment.

In our study, less than half of participants lived in the same city as the physiatrist. Patients with CRPS living in rural areas often shoulder the burdens associated with accessing specialist care in larger urban centers, such as time and cost of travel.^14^ Virtual care addresses a major barrier to care when accessing physicians outside of major urban centers proves challenging.^15^ Although an evaluation of travel logistics was outside the scope of this study, many participants reported benefiting from not having to travel to their appointment.

Early diagnosis and treatment is essential to improving outcomes and quality of life for those experiencing CRPS,^6^ yet the early diagnosis of CRPS is often delayed due to underrecognition and the complexities of the condition.^14^ In our study, most participants found the timeline from injury to referral to appointment to be reasonable. Virtual care may help improve the time from referral to assessment,^14^ though the time from injury to referral depends on physician identification of CRPS symptoms and recognition of the need for referral to an appropriate specialist. We did not formally evaluate whether the timeline from injury to referral or from referral to appointment was decreased with the use of virtual modalities.

A previously documented limitation of virtual care in rehabilitation medicine is the inability to perform a complete physical exam.^2,16^ Some studies assessing patient satisfaction with virtual physical examination for spinal injuries showed that an adequate array of physical exam maneuvers could be performed by the patient—with the help of a family member, if needed—and the findings of our survey suggest that patients are satisfied with these modified physical exams.^12,13^ In our study, the virtual physical exam was modified (e.g., reflexes were not tested) to diagnose the signs of CRPS and was found to be acceptable by the majority of patients. The Budapest Criteria rely on patient-reported symptoms, which are not altered in the virtual context. The majority of patients in this study were satisfied with the virtual physical exam and found it to be comprehensive. Some participants missed the hands-on element of a physical exam, which they felt to be an important factor in guiding treatment. We do note that the great majority had been assessed by a surgeon in person, precipitating the referral. A shortcoming of our study is that patients were not explicitly asked what contributed to their dissatisfaction with the physical exam. It may be that patients perceive a physical exam to be superior when physical contact is included, though the literature on this subject is sparse. In our study, all participants were able to follow the virtual exam directions without issue. These results suggest that the virtual setting was not a barrier to carrying out an appropriate physical exam for the evaluation of CRPS, even when satisfaction with a virtual exam may vary.

The treatment for CRPS includes a regimen of prednisone outlined in the Methods section. Though most participants were satisfied with their treatment, almost half of participants had some concerns about being treated with prednisone. This finding has been observed in another study among patients with rheumatoid arthritis that found that patient perceptions of prednisone were largely negative and were due to misinformation about possible side effects and that perceptions remained somewhat negative posttreatment despite positive outcomes with the treatment.^17^

Hand therapy is an effective adjunct to medical therapy for CRPS.^18^ Virtual hand therapy was available to most participants in our study, before and/or after assessment for CRPS (Table 2). Our qualitative results show that although most patients were satisfied with virtual care, some patients would like practitioners to be able to manipulate their hand for demonstration of activities. One patient recommended recording virtual hand therapy sessions to facilitate activity recall and to track progress.

Resolution of CRPS symptoms is variable depending on treatment modality.^19^ More than half of the participants in our study reported a return of mild to moderate symptoms beyond 4 months after treatment. Return of symptoms may have altered patient satisfaction with their diagnosis and treatment. Total scores had a range in values of over 50 points. Despite this variability, change at the group level was statistically significant. Further, the strong effect size (*d* = 2.2) exceeded that reported using a well-validated condition-specific measure (ES = 1.99) in an observational cohort followed for 1 year after symptom onset.^20^ It is important to note that the relationship between overall recovery and response to CRPS treatment is influenced by the nature and severity of the precipitating injury and that treating CRPS does not guarantee resolution of symptoms due to the initial insult.

Our results show that all patients were comfortable or had neutral feelings about attending a virtual appointment before they had tried it, and no patients felt uncomfortable with trying a virtual appointment. Most patients reported being comfortable telling their story in a virtual setting, and most were happy to share information and to receive health advice virtually. When given the opportunity to provide qualitative feedback on their overall experience with virtual care for CRPS, most participants regarded the virtual experience as positive. Around half of participants would have preferred an in-person appointment if possible, though many of these participants still reported being generally satisfied with the virtual alternative, and many stated that they would recommend virtual care to others.

A limitation of this study is its small sample size of *n* = 13, as well as the possibility that patients who did not complete the survey were experiencing barriers to virtual care. Additionally, we could have asked specific qualitative questions that may have yielded valuable information, such as why some patients would have preferred an in-person appointment over a virtual one or why some patients were not satisfied with the virtual physical exam. Lastly, though our study did not assess other benefits of virtual care, such as financial savings to patients and health care systems, these benefits have been documented in disciplines outside of physical medicine and rehabilitation.^21^ Similarly, our study did not assess barriers that may have prohibited patients from receiving appropriate referral and/or access to physiatry services for CRPS in the first place, such as language, socioeconomic status, and access to WiFi or a device, as seen in other studies assessing access to virtual care.^22^ Future directions could include development of protocols for virtual evaluation and management of CRPS. Additionally, given that this study was conducted for the purposes of quality improvement, the results cannot be generalized to a wider population but could serve to improve the delivery of virtual care for CRPS diagnosis and treatment in our institution.

## Conclusion

In this quality improvement study, no major barriers to engaging in virtual care were identified, and the majority of patients were satisfied with a virtual appointment for evaluation of CRPS. The majority of patients were also satisfied with a decision, based on virtual assessment, to treat CRPS with prednisone. Additionally, though not all patients were fully satisfied with the perceived comprehensiveness of a virtual physical exam to evaluate suspected CRPS, the virtual physical exam was used to diagnose CRPS and was not too challenging for patients to carry out at home with guidance and observation from a provider in real time. The use of virtual appointments helped not only to overcome challenges posed by COVID-19 restrictions but also to address geographic disparities in access to specialist care for CRPS. Results of this study merit further exploration of the use of virtual care as a potentially effective and patient-accepted modality for assessing CRPS.

## Supplementary Material

Supplemental Material

## References

[1] McIntyre M, Robinson LR, Mayo A. Practical considerations for implementing virtual care in physical medicine and rehabilitation: for the pandemic and beyond. Am J Phys Med Rehabil. 2020;99(6):464–67. doi:10.1097/PHM.0000000000001453. 32324617 PMC7253038

[2] Verduzco-Gutierrez M, Bean AC, Tenforde AS, Tapia RN, Silver JK. How to conduct an outpatient telemedicine rehabilitation or prehabilitation visit. PM R. 2020;12(7):714–20. doi:10.1002/pmrj.12380. 32297458

[3] Verduzco-Gutierrez M, Romanoski NL, Capizzi AN, Reebye RN, Kotteduwa Jayawarden S, Ketchum NC, O’Dell M. Spasticity outpatient evaluation via telemedicine: a practical framework. Am J Phys Med Rehabil. 2020;99(12):1086–91. doi:10.1097/PHM.0000000000001594. 32932356

[4] Goh EL, Chidambaram S, Ma D. Complex regional pain syndrome: a recent update. Burn Trauma. 2017;5(1). doi:10.1186/s41038-016-0066-4. PMC5244710 28127572

[5] Ratti C, Nordio A, Resmini G, Murena L. Post-traumatic complex regional pain syndrome: clinical features and epidemiology. Clin Cases Miner Bone Metab. 2015;12(1):11–16. doi:10.11138/ccmbm/2015.12.3s.011. PMC4832405 27134626

[6] Taylor S, Noor N, Urits I, Paladini A, Sadhu MS, Gibb C, Carlson T, Myrcik D, Varrassi G, Viswanath O. Complex Regional Pain Syndrome: A Comprehensive Review. Pain Ther. 2021. doi:10.1007/s40122-021-00279-4. PMC8586273 34165690

[7] de Mos M, de Bruijn AGJ, Fjpm H, Dieleman JP, Stricker BHC, Mcjm S. The incidence of complex regional pain syndrome: a population-based study. Pain. 2007;129(1–2):12–20. doi:10.1016/j.pain.2006.09.008. 17084977

[8] Harden RN, Bruehl S, Perez RSGM, Birklein F, Marinus J, Maihofner C, Lubenow T, Buvanendran A, Mackey S, Graciosa J. Validation of proposed diagnostic criteria (the “Budapest Criteria”) for complex regional pain syndrome. Pain. 2010;150(2):268–74. doi:10.1016/j.pain.2010.04.030. 20493633 PMC2914601

[9] Jamroz A, Berger M, Winston P. Prednisone for acute complex regional pain syndrome: a retrospective cohort study. Pain Res Manag. 2020;2020:1–10. doi:10.1155/2020/8182569. PMC7060858 32184912

[10] Packham TL, MacDermid JC, Michlovitz SL, Buckley N. Content validation of the patient-reported hamilton inventory for complex regional pain syndrome: validité de contenu du hamilton inventory for complex regional pain syndrome, une mesure des résultats déclarés par le patient. Can J Occup Ther. 2018;85(2):99–105. doi:10.1177/0008417417734562. 29475370

[11] Cohen J. Statistical power analysis for the behavioral sciences. New York: Routledge; 1988. doi:10.4324/9780203771587.

[12] Hobson S, Aleem IS, Bice MJ, Butt BB, Bydon M, Elder BD, Fredericks DR Jr, Helgeson MD, Patel RD, Sebastian A, et al. A Multicenter Evaluation of the Feasibility, Patient/Provider Satisfaction, and Value of Virtual Spine Consultation During the COVID-19 Pandemic. World Neurosurg. 2021;154:e781–e789. doi:10.1016/j.wneu.2021.08.004. 34389525 PMC8490082

[13] Bhuva S, Lankford C, Patel N, Haddas R. Implementation and patient satisfaction of telemedicine in spine physical medicine and rehabilitation patients during the COVID-19 shutdown. Am J Phys Med Rehabil. 2020;99(12):1079–85. doi:10.1097/PHM.0000000000001600. 32969967

[14] Katzman JG. Making connections: using telehealth to improve the diagnosis and treatment of complex regional pain syndrome, an underrecognized neuroinflammatory disorder. J Neuroimmune Pharmacol. 2013;8(3):489–93. doi:10.1007/s11481-012-9408-6. 23054372

[15] McGrail KM, Ahuja MA, Leaver CA. Virtual visits and patient-centered care: results of a patient survey and observational study. J Med Internet Res. 2017;19(5):e177. doi:10.2196/JMIR.7374. 28550006 PMC5479398

[16] Reebye R, Finlayson H, May C, Satkunam L, Wein T, Miller T, Boulias C, O’Connell C, Bohorquez A, Dukelow S, et al. Practical Guidance for Outpatient Spasticity Management during the Coronavirus (COVID-19) Pandemic: Canadian Spasticity COVID-19 Task Force. Can J Neurol Sci. 2020;00:1–5. doi:10.1017/cjn.2020.104. PMC7298095 32450934

[17] Van Der Kooij SM, De Vries-Bouwstra JK, Goekoop-Ruiterman YPM, van Zeben D, Kerstens PJSM, Gerards AH, van Groenendael JHLM, Hazes JMW, Breedveld FC, Allaart CF. Limited efficacy of conventional DMARDs after initial methotrexate failure in patients with recent onset rheumatoid arthritis treated according to the disease activity score. Ann Rheum Dis. 2007;66(10):1356–62. doi:10.1136/ard.2006.066662. 17293364 PMC1994290

[18] Zagzoog N, Chinchalkar SJ, Sumsion T. Client satisfaction of hand therapy intervention: an evaluation of the effectiveness of therapy for clients recovered from complex regional pain syndrome. Can J Plast Surg. 2008;16(1):27–35. doi:10.1177/229255030801600103. 19554162 PMC2690627

[19] Misidou C, Papagoras C. Complex regional pain syndrome: an update. Mediterranean Journal of Rheumatology. 2019;30(1):16–25. doi:10.31138/mjr.30.1.16. 32185338 PMC7045919

[20] Packham TL, Bean D, Johnson MH, MacDermid JC, Grieve S, McCabe CS, Harden RN. Measurement properties of the SF-MPQ-2 neuropathic qualities subscale in persons with CRPS: validity, responsiveness, and Rasch analysis. Pain Med. 2019;20(4):799–809. doi:10.1093/pm/pny202. 30346579

[21] Vorenkamp KE. Improving pain care through telemedicine: future or folly? Pain Med. 2016;17(6):997–98. doi:10.1093/pm/pnw035. 26987348

[22] Watts KL, Abraham N. “Virtually perfect” for some but perhaps not for all: launching telemedicine in the bronx during the COVID-19 pandemic. J Urol. 2020;204(5):903–04. doi:10.1097/JU.0000000000001185. 32519903

