# Supplementary material for: Patient satisfaction with virtual evaluation, diagnosis, and treatment of CRPS
Source: Can J Pain. 2022 Jun 3;6(1):78–85. doi: 10.1080/24740527.2022.2063113 (PMC9176228; doi:10.1080/24740527.2022.2063113)
Supplement: Supplemental Material [file UCJP_A_2063113_SM1553.docx]

Supplemental Table 1. PR-HI-CRPS scores

| Variable (n=13) | Time | Mean (/100) standardized score | Standard Deviation | Minimum | Maximum |
| --- | --- | --- | --- | --- | --- |
| Symptoms (18 items) | Baseline | 67.9 | 14.3 | 38 | 91.7 |
|  | Current | 27.4 | 20.2 | 1.8 | 63.9 |
| Impact on daily activities (11 items) | Baseline | 61.5 | 18.9 | 27.3 | 83.3 |
|  | Current | 34.6 | 21.2 | 0 | 71.2 |
| Impact on psychosocial function (11 items) | Baseline | 48.1 | 18.3 | 21.2 | 78.8 |
|  | Current | 19.0 | 16.6 | 1,5 | 62.1 |
| Total score (40 items) | Baseline | 60.5 | 13.3 | 42.1 | 83.3 |
|  | Current | 27.5 | 17.0 | 1.3 | 55.4 |
